# A Case of Merkel Cell Carcinoma of Unknown Primary Origin Presenting With High Merkel Cell Polyomavirus DNA Levels in Swabs Obtained From the Normal Skin

**DOI:** 10.7759/cureus.75837

**Published:** 2024-12-16

**Authors:** Issei Kido, Takao Kadono, Yumiko Hashida, Masanori Daibata, Kozo Nakai

**Affiliations:** 1 Dermatology, Kochi University, Nankoku, JPN; 2 Microbiology and Infection, Kochi University, Nankoku, JPN

**Keywords:** merkel cell carcinoma, merkel cell polyomavirus, normal skin, swabs, unknown origin

## Abstract

Merkel cell carcinoma is a rare neuroendocrine tumor with high mortality. It is well known that clonal integration of the Merkel cell polyomavirus into the dermal precursor cells is a hypothesized pathway in Merkel cell carcinoma pathogenesis. Here, we demonstrate a case of Merkel cell carcinoma (primary origin unknown) presenting with high Merkel cell polyomavirus DNA levels in swabs obtained from normal skin.

## Introduction

Merkel cell carcinoma (MCC) is an uncommon neuroendocrine tumor with high mortality. MCC usually occurs on the skin of the head, neck, and extremities [1]. However, approximately 4% of all MCCs present without skin involvement or a history of primary cutaneous origin [2]. It is well known that clonal integration of the Merkel cell polyomavirus (MCPyV), a double-stranded DNA virus of the Polyomaviridae family, into the dermal-located precursor cells is a hypothesized pathway in MCC pathogenesis [3]. MCPyV has been reported to be found in 70-80% of cases of MCC [4,5]. Although the oncogenic role of MCPyV has been noted, this viral DNA can be found in low copy numbers in normal skin of healthy individuals [6]. Therefore, MCPyV might be a kind of the normal microbiome of human skin, and how this virus is transformed into an oncogenic form remains unclear. Considering the infrequent prevalence of MCC compared with the finding of MCPyV in normal skin, there might be meaning in clarifying high-risk groups for the onset of MCPyV-positive MCC among healthy individuals.

A previous report revealed a specific group of healthy people who had high levels of MCPyV infection on their sun-exposed skin [7]. In addition, higher MCPyV DNA levels were shown in swabs taken from normal skin surfaces of patients with MCC (primary origin known) compared with those from healthy individuals [8]. These studies suggest that MCC occurs under the background of high MCPyV-loaded skin, and are predicted to stimulate further studies on whether the levels of such cutaneous virome could be one of the markers for the onset of MCC.

Here, we demonstrate a case of MCC (primary origin unknown) presenting with high MCPyV DNA levels in swabs obtained from normal skin.

## Case presentation

**Figure 1:**
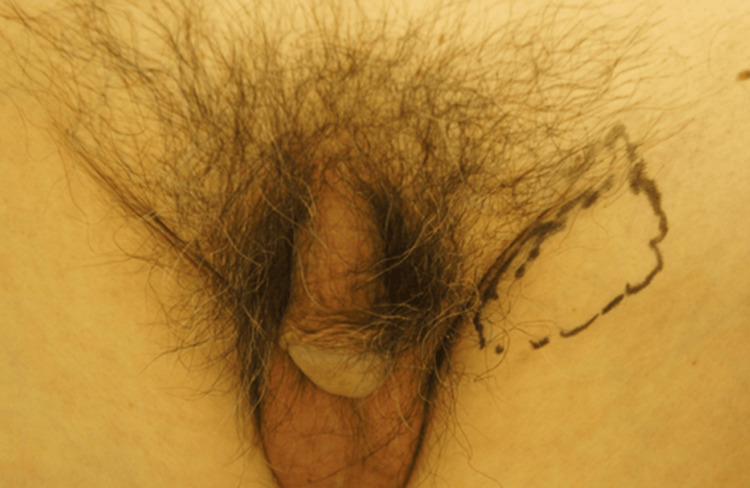
Clinical presentation. An expanding subcutaneous tumor was found in the left inguinal area.

An 82-year-old male was referred to our department with an expanding subcutaneous tumor in the left inguinal area (Figure 1).

**Figure 2:**
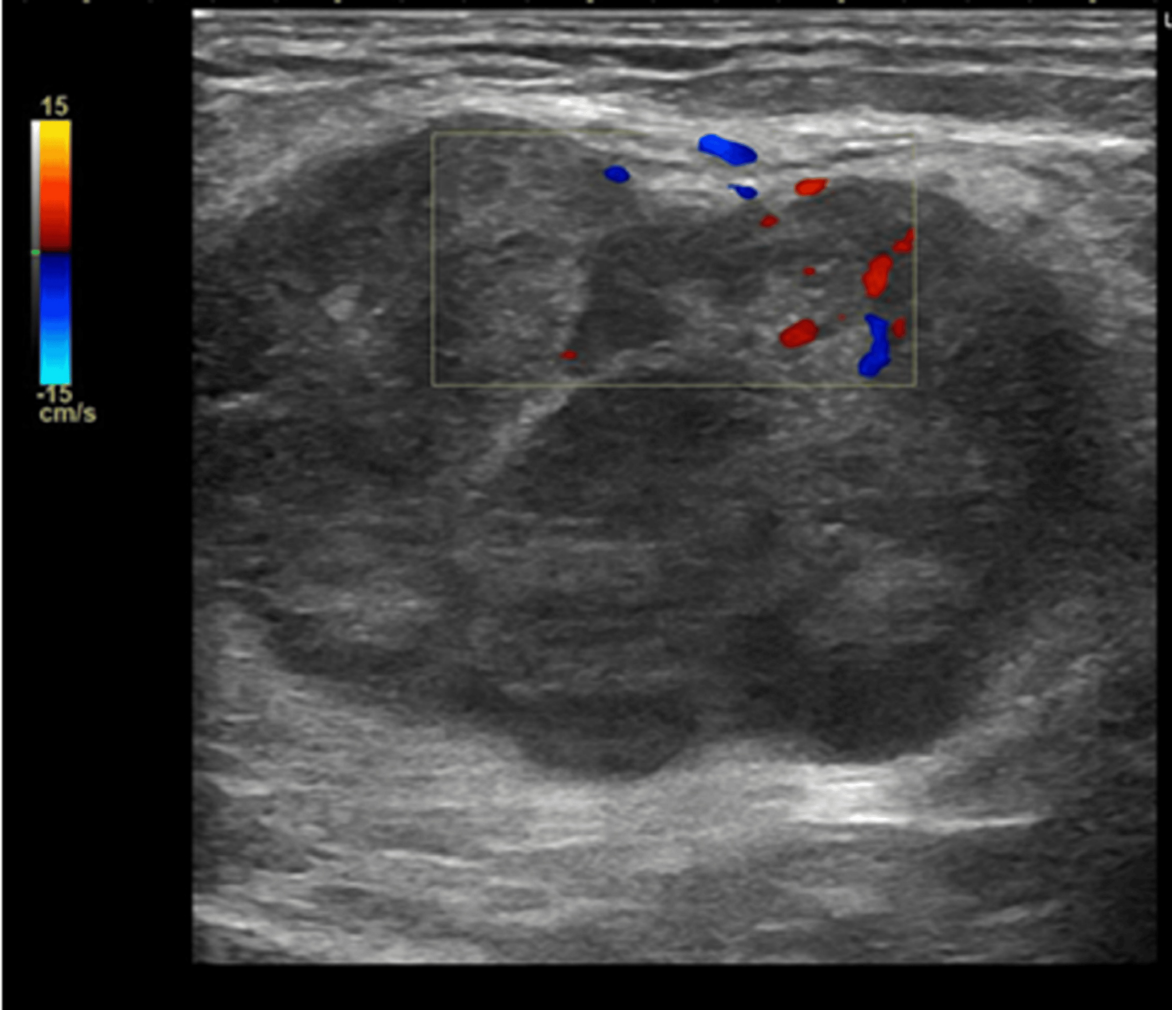
Ultrasonography. A 35 mm hypoechoic mass was observed in the subcutaneous fat layer.

Ultrasonography revealed a 35 mm hypoechoic mass in the subcutaneous fat layer (Figure 2).

**Figure 3:**
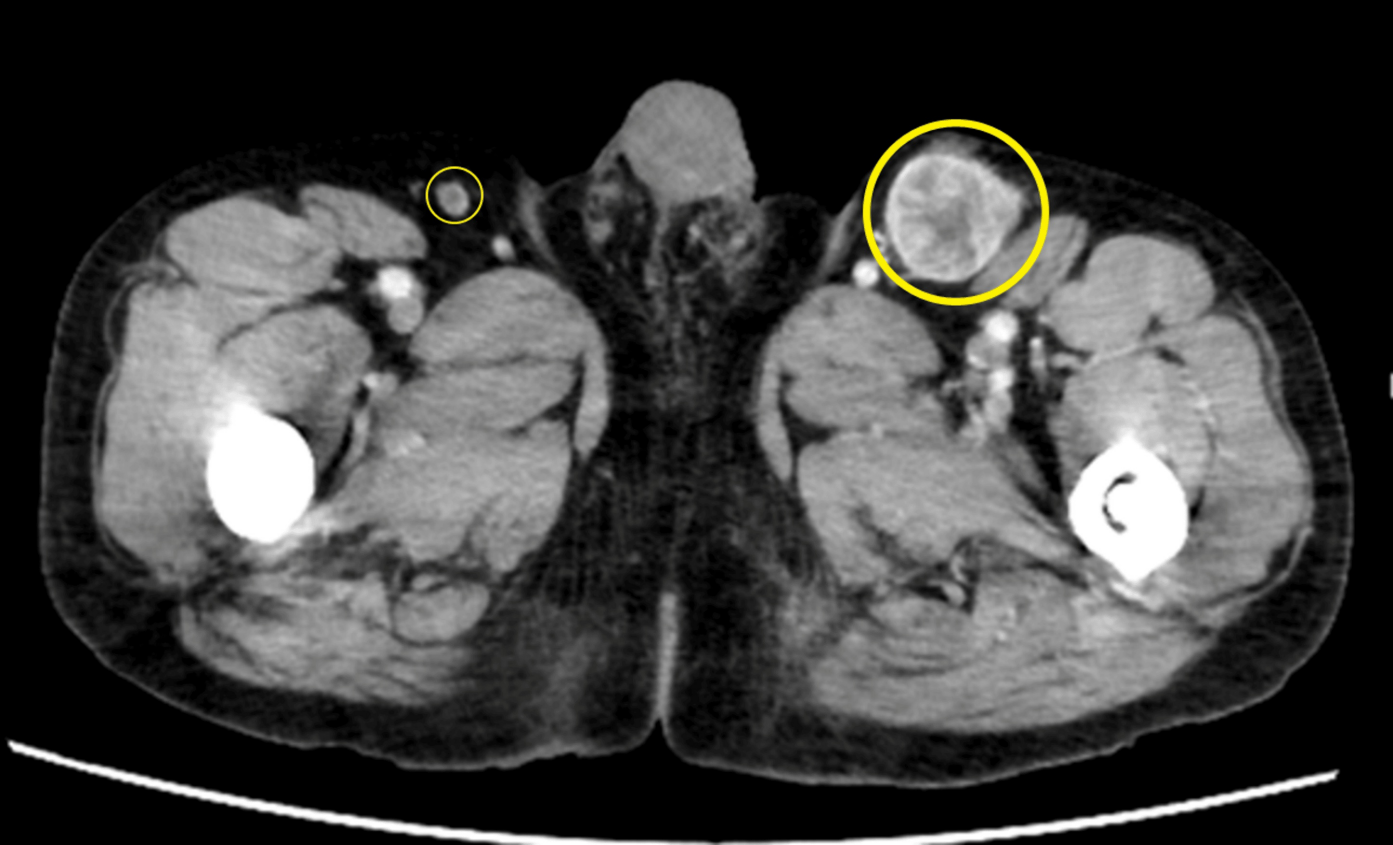
Computed tomography. A partially contrasted mass in the left inguinal area and a small mass (7 mm) were observed in the right inguinal area.

Computed tomography demonstrated a partially contrasted mass in the left inguinal area and a small mass (7 mm) in the right inguinal area (Figure 3).

**Figure 4:**
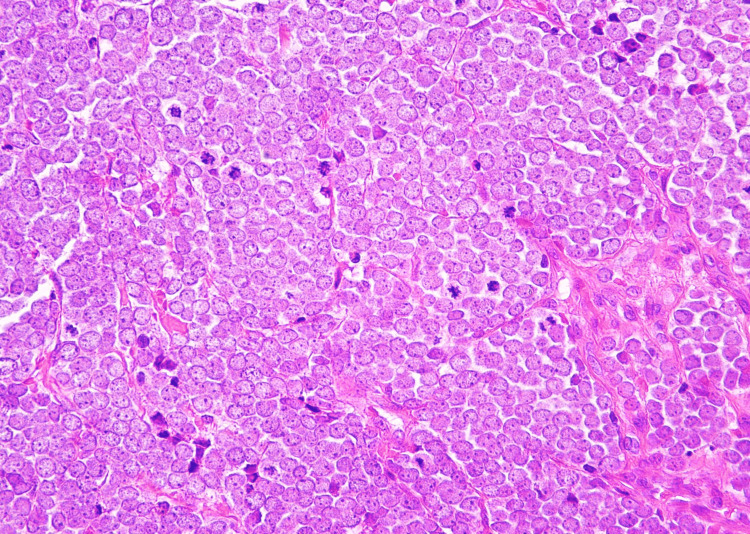
Histology (Hematoxylin and eosin, ×200). A dense population of oval-shaped basophilic cells with nuclear molding and mitotic figures was observed in the lymph node.

Histological examination of the swollen lymph node in the left inguinal region revealed a dense population of oval-shaped basophilic cells with nuclear molding and mitotic figures in the lymph node (Figure 4).

**Figure 5:**
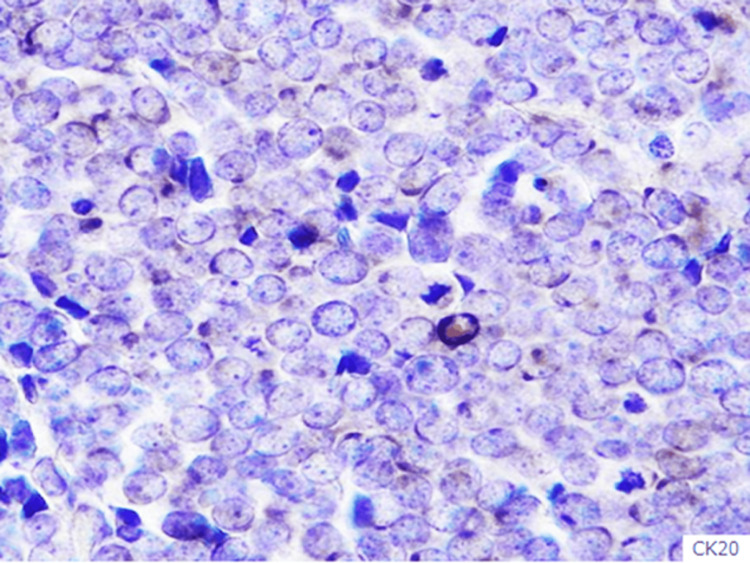
CK20 staining (×400). CK20-positive cells were found.

**Figure 6:**
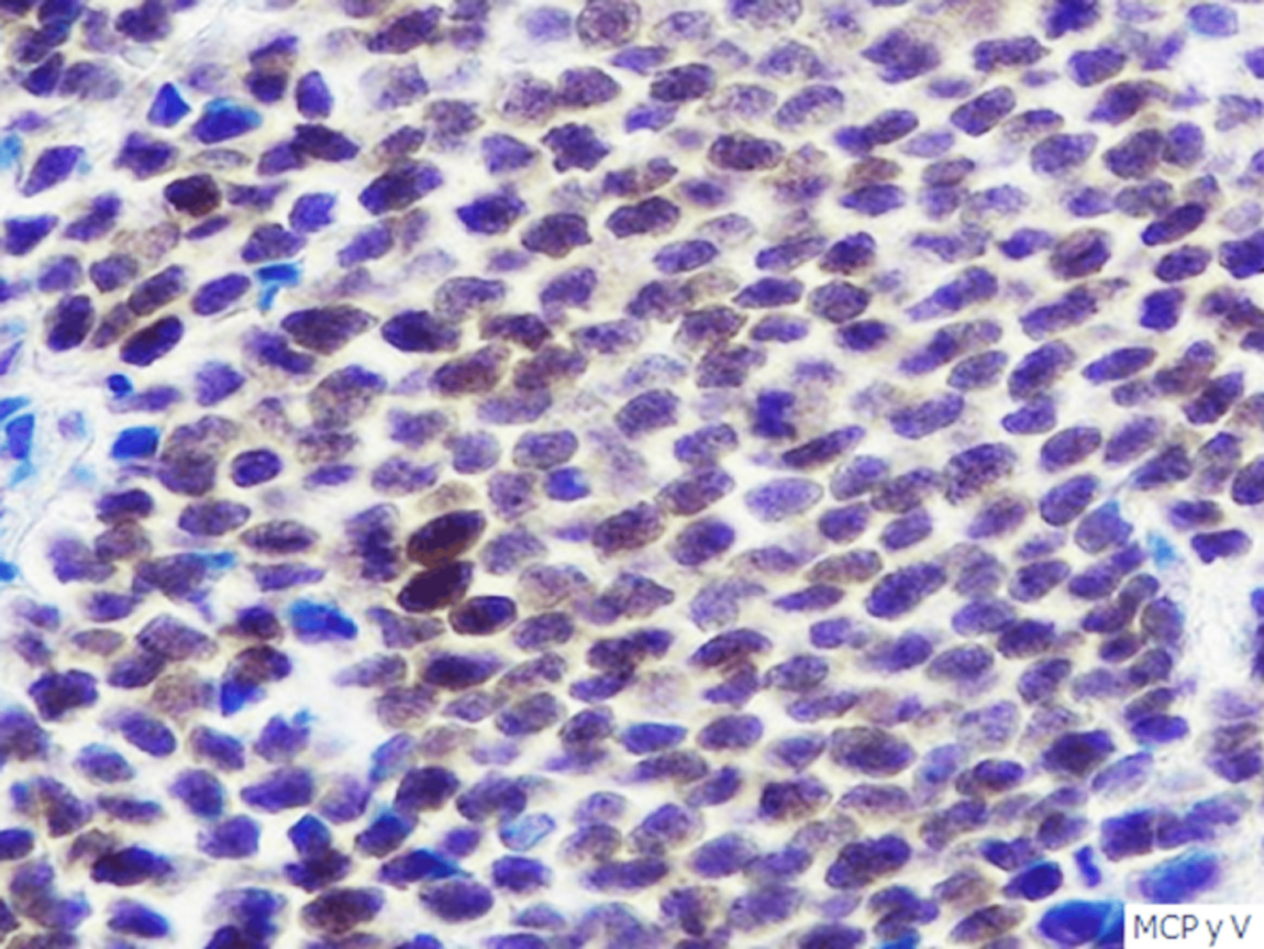
MCPyV staining (×400). MCPyV-positive cells were found. MCPyV: Merkel cell polyomavirus

The immunohistological study demonstrated CK20-positive cells and MCPyV large T-antigen-positive cells (Figures 5-6).

We could not find other MCC-suggested lesions by positron emission tomography. To examine the MCPyV DNA levels of the normal skin surface, skin swabs were obtained from the head, back forearms, thighs, legs, and ankles. Higher levels of MCPyV DNA were detected in swabs taken from the normal skin surface of the patient compared to those from previously reported healthy individuals (Table 1) [8]. 

**Table 1:** Results of MCPyV DNA levels MCPyV DNA levels (copies/ng) in swabs obtained from the normal skin of our case and those from previously reported healthy control/patient. MCC: Merkel cell carcinoma; MCPyV: Merkel cell polyomavirus

Lesion	In This Case: MCC Unknown Primary Origin	Previously Reported Healthy Control (Mean)	Previously Reported MCC Known Primary Origin (Mean)
Head	457.68	263	98268
Back	12.39	Not applicable	Not applicable
Arm (left)	30.22	2.84	133
Arm (right)	34.03	Not applicable	Not applicable
Thigh (left)	8.28	Not applicable	Not applicable
Thigh (right)	26.15	Not applicable	Not applicable
Ankle (left)	9.91	Not applicable	Not applicable
Ankle (right)	3.36	Not applicable	Not applicable

After the diagnosis was confirmed, a total of 66 Gy of radiation therapy was administered to both inguinal lymph nodes. Following the completion of radiation therapy, the left inguinal lymph node, initially measuring 35 mm, was reduced to 17 mm but remained persistent, leading to surgical excision. At 19 months after the initial visit, pulmonary metastases were detected, and 65 Gy of radiation therapy was performed. At 30 months after the initial visit, recurrence was observed in both inguinal lymph nodes, and treatment with avelumab was initiated, which is currently ongoing.

## Discussion

The overall 5-year survival rate is about 50% in patients with MCC disease, and it is less than 20% in patients with metastatic cases [2]. There are a few reports of patients with MCC arising in the lymph nodes without an identified site of cutaneous malignancy [9,10]. These MCCs arising without a primary cutaneous lesion are called MCCs of unknown primary origin [9]. Due to the limited reports of this rare type of MCC, it is believed to be difficult to describe patterns in presentation, pathology, and treatment response, although it is reported that MCC (primary origin unknown) showed an improved prognosis compared to MCC (primary origin known) [11].

The cellular origin of MCC and the pathological roles of MCPyV, especially in the case of MCC (primary origin unknown), still remains unclear [9]. Moreover, the association with MCPyV even in the lesion of MCC (primary origin known) was controversial: high or low [12,13]. Therefore, it might be a challenge to investigate the MCPyV DNA levels in swabs taken from the normal skin surface of patients with MCC. Following the previous study of MCC (primary origin known), to the best of our knowledge, this is the first case of MCC (primary origin unknown) presenting with MCPyV DNA levels in swabs obtained from normal skin.

Although the MCPyV DNA levels were not as high as those of MCC (primary origin known), they were apparently higher than those of healthy controls (Table 1) [8]. We examined the swab sample from the normal skin of various body parts to identify the origin of MCC in our patient. However, the MCPyV DNA levels in swabs from the thigh were not as high as those from the head. These results indicate that the MCPyV DNA levels in swabs could not point out the existence of MCC mass in the inguinal area.

## Conclusions

We demonstrate a case of MCC (primary origin unknown) presenting with high MCPyV DNA levels in swabs obtained from normal skin. Since this is a case report and the previously reported number of patients with MCC (primary origin known) was small, it may be difficult to clarify the meaning of MCPyV DNA levels in swabs obtained from the normal skin, and further reports and/or studies are desired.
